# Comparative studies between a new human rhabdomyosarcoma cell line, JR-1 and its tumour of origin.

**DOI:** 10.1038/bjc.1986.155

**Published:** 1986-07

**Authors:** J. Clayton, J. R. Pincott, J. A. van den Berghe, J. T. Kemshead

## Abstract

**Images:**


*Br. J. Cancer* (1986) **54**, 83–90

### Comparative studies between a new human rhabdomyosarcoma cell line, JR-1 and its tumour of origin

J. Clayton[1], J.R. Pincott[2], J.A. van den Berghe[3] & J. T. Kemshead[1]

[1]*ICRF Oncology Laboratory, Institute of Child Health, London WC1;* [2]*Department of Histopathology, Hospital for Sick Children, Great Ormond Street, London WC1 and* [3]*Department of Paediatric Genetics, Institute of Child Health, London WC1, UK.*

**Summary** A new human embryonal rhabdomyosarcoma cell line, designated JR-1, is described that closely resembles the tumour from which it was derived. Comparative studies, by light and electron microscopy reveal morphological features such as myofibre formation, that are concordant with embryonal rhabdomyosarcoma. Immunohistological investigations using a panel of monoclonal antibodies indicate that the cell surface antigen profile of the JR-1 cell line is similar to other embryonal rhabdomyosarcomas. In addition the cell line expresses the cytoplasmic intermediate filament protein desmin, only found in cells of rhabdoid origin. Karyotyping JR-1 shows the cells to contain variable numbers of chromosomes (range 44–100). DNA flow cytometry indicates that cells have an DNA content which is approximately twice normal. The JR-1 cell line has a doubling time of 29 h in culture and, in common with several other human cell lines, produces xenografts in nude mice within 6 weeks of inoculation. With detailed studies on the original tumour and the JR-1 cell line, the latter should prove an excellent model system for investigating the biology of rhabdomyosarcoma.

Rhabdomyosarcoma is the commonest sarcoma in childhood, accounting for between 4 and 8% of malignant tumours in patients under 15 years of age (Sutow *et al.*, 1984). The most common histological type of childhood rhabdomyosarcoma is the embryonal variety. This consists mainly of small cells, with round to oval nuclei and little cytoplasm, and resemble the primitive mesenchymal stem cell from which the tumour is thought to arise (Enzinger & Weiss, 1983).

By conventional histological and cytological techniques it is sometimes difficult to distinguish rhabdomyosarcomas from other 'small round cell tumours' of childhood, namely lymphoblastic leukaemia/lymphoma, neuroblastoma and Ewing's sarcoma (Reynolds *et al.*, 1981; Triche & Askin, 1983). Whilst it is relatively simple to differentiate neuroblastoma from lymphoblastic leukaemia/lymphoma using panels of monoclonal antibodies, this is not the case for either Ewing's sarcoma or rhabdomyosarcoma (Kemshead *et al.*, 1982). Few *in vitro* cell lines are available to help in the search for new reagents that can selectively identify rhabdomyosarcomas from other 'small round cell' tumours of childhood (McAllister *et al.*, 1969, 1975; Giard *et al.*, 1973). We have systematically attempted to establish new rhabdomyosarcoma cell lines from biopsy and autopsy material. Here we describe one such cell line, JR-1, and demonstrate that both its morphological appearance and immunophenotype are analogous to the tissue from which it was derived.

Correspondence: J.T. Kemshead.
Received 20 December 1985; and in revised form, 20 March 1986.

### Materials and methods

### *Case report*

The JR-1 cell line was established from tumour material obtained at post mortem examination of a 7 year old girl. The patient first presented at the Hospital for Sick Children, Gt. Ormond Street, London, in May 1982, with a uterine neoplasm, following 2–3 weeks of abdominal pain. Biopsy revealed a tumour histologically classified as embryonal rhabdomyosarcoma, believed to have arisen from the left broad ligament. A computerised tomography scan showed metastatic spread of the tumour to the lungs. Following a regimen of radiotherapy with vincristine, cyclophosphamide and Actinomycin D, both the metastases and the primary pelvic mass were notably diminished. In January 1983 lung metastases of increasing size were again observed, and the patient died in May. Post mortem examination revealed a large, partially necrotic, mass of primary tumour filling the pevlis, and widespread, large metastases with no obvious necrosis in the lungs, liver, adrenal, kidneys, pancreas, rectum, bladder and peritoneum. Histological assessment revealed the tumour to be a poorly differentiated embryonal rhabdomyosarcoma.

© The Macmillan Press Ltd., 1986

*Establishment of the JR-1 cell line*

The tissue culture medium used throughout was RPMI 1640 medium, supplemented with 10% foetal calf serum (Gibco Europe Ltd.). 2 mM glutamine (Gibco), 100 IU penicillin (Gibco) and 100 $\mu$g ml$^{-1}$ streptomycin (Gibco).

A specimen of tumour taken from a lung metastasis was washed in fresh medium and chopped into 2–3 mm pieces. These were distributed between 25 cm$^2$ plastic tissue culture flasks (Falcon) containing 5.0 ml of the above medium, and incubated at 37°C in a 5% $CO_2$ incubator.

After 1 week, half the culture medium was replaced. Once sufficient cell growth had occurred complete medium changes were made 3 times a week. On confluency, cells were removed from the dishes using trypsin/versene and subcultured 1:3 into new culture flasks. Cultures were frozen at intervals in medium containing 10% foetal calf serum (FCS) and 10% dimethyl sulphoxide (DMSO) and stored in liquid nitrogen. Human neuroblastoma cell lines with the prefix CHP were obtained from Dr A.E. Evans, The Children's Hospital, Philadelphia, and those with the prefix SK from Dr L. Helson, Sloane Kettering Institute, New York. Lan-1 was obtained from Dr R. Seeger, University of California, Los Angeles. TR14 from Dr T. Rupniack, Imperial Cancer Research Fund, London and 'Kelly' from Dr F. Gilbert, Mount Sinai School of Medicine, New York. All of these lines were grown in the above medium and screened for antibody binding in the logarithmic phase of growth.

*Histological and ultrastructural studies*

Tumour tissue was either fixed in 10% buffered formalin for paraffin wax embedding or snap frozen in liquid nitrogen and stored at $-70$°C. Paraffin wax embedded tissue sections (5 $\mu$m) were cut and stained with haematoxylin and eosin. Frozen sections (5 $\mu$m) were mounted on glass slides, air dried and fixed in methanol/acetone (50/50, v/v) for 2 min at $-20$°C.

Cells grown on coverslips were stained with H&E for morphological examination. For indirect immunofluorescence studies, cytocentrifuged preparations of cells were air dried and fixed in methanol/acetone as described above. Cells ($2 \times 10^6$) for electron microscopy were centrifuged at 350 *g* to form a pellet and fixed in 2.5% glutaraldehyde in 0.1 M cacodylate buffer for 18 h at 4°C. The pellet was washed twice in cacodylate buffer and secondarily the pellet was again fixed in 1% osmium tetroxide in cacodylate buffer. After washing and dehydration using alcohol and propylene oxide, samples were embedded in Epon resin (Agar Aids, Essex, U.K.). Ultra-thin sections were examined under a Joel 100c transmission electron microscope.

*Cytochemical and immunohistological procedures*

Tissue sections and cytocentrifuge preparations were stained with phosphotungstic acid haematoxylin (PTAH) to demonstrate myofibres. Samples were also treated with periodic acid-Schiff (PAS) staining to detect glycogen granules.

For indirect immunofluorescence assays on cell lines $5 \times 10^5$–$10^6$ cells were incubated for 30 min at 4°C, with an excess of each monoclonal antibody listed in Table I. Following washing, antibody binding was demonstrated using fluorescein conjugated goat anti-mouse immunoglobulin (Ig), that was affinity purified and cross absorbed against human Ig and pig liver powder. Samples were analysed using a Zeiss Photomicroscope III with epi-illumination optics.

*Chromosome analysis*

JR-1 cells were accumulated at metaphase by the addition of 1 $\mu$g ml$^{-1}$ of vinblastine for 1 h during the logarithmic phase of growth. Adherent cells were detached from the flasks by trypsin/versene treatment, washed and resuspended in 0.075 M KCl for 20 min. Following fixation in 3 successive washes of 3:1 methanol:acetic acid, cells were dropped onto microscope slides and flame dried.

G-banding was achieved by a one minute incubation of slides in Hanks balanced salt solution (HBSS) $-Ca^{++}$, $-Mg^{++}$ (HBSS, pH 6.8) and 20 mg ml$^{-1}$ trypsin in HBSS pH 6.7. This was followed by immersion in HBSS (pH 5.7), a 4 min incubation in 5% Giemsa buffer (pH 6.8) and washing in deionized water.

### Results

*Tumour histology*

Staining of the original uterine tumour revealed mostly undifferentiated pleomorphic cells. Occasional spindle and strap-shaped cells and some multinucleated giant cells with strongly eosinophillic cytoplasm were identified. Cells contained round, oval or indented nuclei with prominent nucleoli. Occasionally cytoplasmic longitudinal fibres and cross striations were seen, suggesting a diagnosis of embryonal rhabdomyosarcoma. Primitive myofibres could be demonstrated within the more differentiated tumour cells by PTAH staining. Fine, but not abundant, glycogen granules were found to be present by PAS staining.

**Table I** Monoclonal antibodies used in the characterisation of the JR-1 cell line

| *Antibody* | *Immunogen* | *Antigen* | *Reference* |
|---|---|---|---|
| Desmin | Commercial | Desmin | Debus (1983) |
| Vimentin | Commercial | Vimentin | Osborn (1984) |
| Myoglobin[e] | Commercial | Myoglobin | Dako Products Ltd |
| FD 19.9 | Human astrocytoma | GFAP[a] | Kemshead & Coakham (1983) |
| LE 61 | Epithelial cells | Cytokeratin | Lane (1982) |
| RT 97 | Rat brain extract | Neurofilament | Wood & Anderton (1981) |
| UJ13A | Human foetal brain | Glycolipid | Allan *et al.* (1983) |
| UJ181.4 | Human foetal brain | NK[b] | Kemshead *et al.* (1983) |
| UJ 127.11 | Human foetal brain | 220,000 Mol.wt Gp. | Kemshead *et al.* (1983) |
| UJ308 | Human foetal brain | NK | Kemshead *et al.* (1982) |
| A2B5 | Chick retinal cells | Ganglioside Gq | Eisenbarth *et al.* (1979) |
| Thy-1 | Purified Thy-1 | Thy-1 | Kemshead *et al.* (1982) |
| PI 153/3 | Neuroblastoma cells | p 20,000 Mol.wt | Kennett & Gilbert (1979) |
| 5.1.H11 | Myoblasts culture | NK | Hurko & Walsh (1983) |
| BA1 | Nalm6-M1-ALL cells | NK | Abramson *et al.* (1981) |
| BA2 | Nalm6-M1-ALL cells | p 24,000 Mol.wt[c] | Kersey *et al.* (1981) |
| BA3 | Nalm6-M1-ALL cells | 95,000 Mol.wt.Gp[d] | Le Bien *et al.* (1982) |
| W6/32 | Human leucocytes | Gp 44 & 12,000 | Brodsky *et al.* (1979) |
| DA2 | RKT Lymphoid line | DR antigens | Brodsky *et al.* (1980) |
| 2D1 | Human leucocytes | 2000,000 Mol.wt Gp | Beverley (1980) |

[a]Glial Fibrillary Acidic Protein; [b]Not known; [c]Protein; [d]Glycoprotein and [e]Heteroantisera.
Anti-Desmin was purchased from Amersham Int. PLC; Vimentin was purchased from Eurodiagnostics and Myoglobin was purchased from Dako Products.

*Histology of cultured cells*

The cell line JR-1 shows pleomorphic histological features similar to the tumour of origin with considerable variety in the size and shape of cells. Stellate cells predominate, whilst elongated, spindle-, strap-shaped, rounded, oval and tadpole-shaped cells can be identified (Figure 1). In addition a small number of large multinucleated cells are present ~1.0%).

In this population nuclei vary in shape between round, oval and indented, and contain coarse chromatin and either very prominent single or multiple nucleoli (Figure 2A). The large multinucleate cells have nucleoli, arranged either in tandem or in a horseshoe distribution. The cytoplasm of JR-1 cells shows fine stippled granules staining positive with PAS, which disappear on diastase digestion. This demonstrates the presence of glycogen granules. Moderate numbers of mitotic figures are present. The ample cytoplasm appears granular and highly eosinophilic, although there is considerable variation in the intensity of H&E

**Figure 1** JR-1 cell line, morphology of individual cells. (a) stellate, (b) strap-shaped, (c) spindle, (d) large multinucleated giant cell, (e) elongated cell with the nuclei arranged in tandem. (Haematoxylin and eosin, ×378).

**Figure 2** (A) Toluidine blue-stained semi-thin section, illustrating nuclear morphology characteristic of the JR-1 cell line. (1) multinucleated giant cell containing nuclei arranged in horseshoe distribution with prominent nucleoli; (2) cell with one round and one indented nucleus x nucleoli are prominent. ( x 600). (B) Electron micrograph showing focal condensations of cytoplasmic myofibres suggestive of Z bands. N = nucleus, CY = cytoplasm. ( x 20,000)

staining. PTAH staining shows the presence of longitudinal fibrils and transverse striations (see Figure 3 A–C).

*Electron microscopy*

Electron microscopy of the JR-1 line shows rounded cells containing nuclei with granular chromatin, and an abundant cytoplasm rich in organelles (see Figure 2B).

The cytoplasm contains many mitochondria and in some cells dilated cisternae are identified. In concordance with the PAS staining, seen by light microscopy, small numbers of cytoplasmic glycogen granules are identified together with free ribosomes and rough endoplasmic reticulum. Thick and thin filaments are found in the majority of cells. These show a relationship characteristic of actin and myosin, both on longitudinal and transverse section. In some areas fibres appear condensed, as is seen in *Z* band formation (Figure 2B). There is no evidence of epithelial features. In particular, the cytoplasmic condensations lack the triplets of fused microtubules seen in the basal bodies of cilia.

*Growth kinetics*

Cells taken at passage 44 and seeded into 15 mm diameter dishes at an initial concentration of either 10 or $3 \times 10^4$ cells/well exhibited exponential growth with a doubling time of $\sim$29 h (data not presented).

**Figure 3** A, B and C show 3 separate fields of JR-1 cells stained with phosphotungstic acid haematoxylin. Arrows indicate cytoplasmic cross-striations (cs). ( x 600)

*Cytogenetics*

Flow cytometry of mithromycin stained JR-1 cells was undertaken to estimate DNA content. Peripheral blood lymphocytes were used to calibrate the flow cytometer. These gave a fluorescence peak at window 26 representative of normal diploid cells (Figure 4). In contrast the JR-1 cell line gave peak fluorescence at window 48 indicative of a DNA content of approximately twice normal.

Direct counting of 50 cells (passage 45) revealed a chromosome number of between 44 and 100 (with a modal number of 88). A low number of 'double dots' resembling interstitial deletions (Savage, 1975) were found in 10% of these cells.

G-banded karyotypic analysis was carried out on 10 cells, none of which were found to be identical. The majority of chromosomes demonstrated a normal banding pattern, no obvious Y chromosome was present. Eighty percent of cells carried at least one 'marker' chromosome of rearranged material; $13q^-$ occurred in 50% cells, often as an homologous pair, $1p^-$ and der (10) occurred in 30% cells and $1q^-$, $6q^-$ and iso(11q) in 20%. Despite the near tetraploid chromosome number, normal chromosomes varied between cells from 0–8 copies.

**Figure 4** Flow cytometric analysis of mithromycin-stained JR-1 cells produces a main G0/G1 peak, representing a mean DNA content of approximately twice normal, compared with the peripheral blood lymphocyte (PBL) control.

*Establishment of a xenograft in nude mice*

Injection of $5 \times 10^6$ cells s.c. into the flank of nude mice produced tumour nodules at the site of injection within 2 months of inoculation. The morphological and immunological studies on these tumours are in concordance with the JR-1 cell line and the original tumour.

*Immunohistological characteristic of the JR-1 cell line*

Indirect immunofluorescence studies were undertaken with a variety of monoclonal antibodies to characterise fully the JR-1 cell line. Using a panel of antibodies to intermediate filaments 20–30% of the cells were shown to bind anti-desmin, whereas almost all cells bound an anti-vimentin monoclonal antibody (Table II). This pattern of binding was identical to that seen on frozen sections of the tumour from which JR-1 was derived. In both cases no binding of antibodies to glial fibrillary acidic protein (GFAP), neurofilaments or cytokeratin was detected. Eleven other rhabdomyosarcomas also demonstrated a similar pattern of intermediate filament expression following immunohistological analysis. No binding of an anti-myoglobin antibody was observed in either the JR-1 line or tumour tissue, a finding

**Table II** Cytoplasmic antigen expression in rhabdomyosarcoma tumours and the JR-1 cell line

| | *Anti desmin* | *Anti vimentin* | *Anti GFAP (FD 19.9)* | *Anti cytokeratin* | *Anti neurofilament RT97* | *Anti myoglobin* |
|---|---|---|---|---|---|---|
| JR-1 line | +[a] | + | — | — | — | — |
| JR-1 tumour | +[a] | + | — | — | — | — |
| Other rhabdomyosarcomas | 11/11 | ND | 0/11 | 0/11 | 0/11 | ND |

[a]Positive in 20–30% of cells.
ND—No data.

consistent with the relatively undifferentiated nature of the original tissue.

Table III shows the profile of cell surface antigens expressed by the JR-1 cell line when examined using a panel of monoclonal antibodies. The antibody W6/32, recognising a monomorphic determinant expressed on HLA ABC antigens, was found to bind to both the cell line and tumour tissue. In contrast no binding of DA2, an anti-DR antibody, was detected. No other lymphoid markers (2D1, PI 153/3, BA1, BA2, BA3) bound to the JR-1 cell line. BA2 has been shown to bind to other 'small round cell' tumours of childhood, *viz* some neuroblastoma and Ewing's sarcoma cell lines. However, some antibodies raised against neural tissue (A2B5, UJ13A) and some others selected for their binding to human neuroblastoma (anti Thy-1, 5.1.H11) bound to the JR-1 cell line. Although there is some heterogeneity in antigen expression among the 12 human neuroblastoma lines studied, the pattern of antibody binding to the JR-1 rhabdomyosarcoma line differs from neuroblastoma (Table III).

### Discussion

The pancity of human rhabdomyosarcoma cell lines described to date, reflects the rare occurrence of the tumour and the difficulty of establishing *in vitro* lines from childhood solid tumours. It is particularly important to establish cell lines of this tumour to investigate further its biology. Hopefully this will lead to an increase in the survival rate of children with this malignancy. The JR-1 cell line described in this report resembles, as far as we can determine, the tumour from which it was derived. In addition, the morphology of this cell line is similar to the previously published embryonal rhabdomyosarcoma derived lines RD618, TE441 and the rhabdomyosarcoma line RD114 (McAllister, 1972). Of these, only JR-1 and RD618 show cytoplasmic banding following PTAH staining, a feature supporting their rhabdoid derivation.

Classification of tumours is possible by intermediate filament typing (Osborn & Weber, 1983). The pattern of intermediate filament expression shown by JR-1 and other rhabdomyosarcomas differs from that seen in other 'small round cell' tumours of childhood. Neuroblastomas, particularly those showing a degree of neural differentiation, may contain neurofilaments, whereas Ewing's sarcoma and lymphoid malignancies contain vimentin. Desmin has proved useful in distinguishing sarcomas of muscle cell origin from other soft tissue sarcomas, and from childhood tumours of different derivation (Attmansbergen *et al.*, 1985). Our studies show that over 20 samples of fresh neuroblastoma and 11 of Ewing's sarcoma do not bind monoclonal anti-desmin. Desmin was found in the JR-1 cell line and in the rhabdomyosarcoma cell line RD618. In

**Table III** Binding of a panel of monoclonal antibodies to the JR-1 cell line and the tumour from which it was derived

| *Monoclonal antibody* | *JR-1 rhabdomyosarcoma cell line* | *JR-1 original tumour tissue* | *Other rhabdomyosarcoma tumours*[a] | *Human neuroblastoma cell lines*[b] |
|---|---|---|---|---|
| UJ13A | + | + | 11/11 | 11/11 |
| UJ181.4 | — | — | 1/9 | 9/11 |
| UJ127.11 | — | — | 1/6 | 10/11 |
| UJ308 | — | — | 2/9 | 5/10 |
| A2B5 | + | + | 5/6 | 10/11 |
| X Thy-1 | + | + | 4/5 | 9/11 |
| PI 153/3 | — | — | 3/6 | 11/11 |
| 5.1.H11 | + | + | 11/11 | 11/11 |
| BA1 | — | ND | 0/3 | 11/11 |
| BA2 | — | ND | ND | 11/11 |
| BA3 | — | — | 0/4 | 0/8 |
| W6/32 | + | + | 2/3 | 6/11[c] |
| DA2 | — | — | 0/3 | 0/11 |
| 2D1 | — | — | 0/6 | 0/11 |

[a]All tumours used in this study were classified as embryonal rhabdomyosarcomas according to the clinical features of the disease, histological and cytological studies and immunological characterisation with anti-desmin and myoglobin; [b]CHP 100, CHP 212, CHP 126, CHP 134, Lan-1, TR14, Kelly, SK-N-SH, SK-N-BE, SK-N-FL, SK-N-D2 and [c]Weak binding in the majority of cases. ND—No data.

contrast, no binding was seen to rhabdomyosarcoma derived cell lines A673 or A204. Neither did A673 stain with antibody A2B5, or A204 with UJ13A. Despite JR-1 binding A2B5, UJ13A and other antibodies described as being 'neuroectodermal' in specificity, the line is unquestionably rhabdoid in nature. In addition to the data on rhabdomyoblast-specific cell surface and intracellular antigens (Tables II–III), particles and cytoplasmic banding are seen on PTAH staining (Figure 2). Electron microscopy also reveals thick and thin filaments and a tendency for Z bands to form (Figure 3). These are definitive criteria for the diagnosis of rhabdomyosarcoma (Toker, 1968). In support of the proposed origin of JR-1, the clinical presentation of this tumour arising in the left broad ligament is not a recognised site of occurrence of neuroblastoma.

Evidence of the establishment of JR-1 as a cell line comes from continual growth of cells in culture (currently to passage 58) and the ability of cells to grow as a xenograft in nude mice (cells taken at passage 44).

Unfortunately G banding and karyotypes of JR-1 has not revealed a consistent chromosomal marker associated with the line. No consistent marker has been described in rhabdomyosarcoma in the past, although an increase in abberrations of chromosome 3 has been suggested as being linked with the malignancy. Despite culture for over 50 passages considerable variation in chromosome number persists within the line. Despite this variation, the growth rate determined between passages 32–50 has remained constant and the expression of cytoplasmic and cell membrane antigens within the cell line is relatively consistent from cell to cell. This suggests the heterogeneity in chromosomal number is due to the instability of near tetraploid cells, rather than due to several contaminating cell populations. Currently the line is being cloned to determine whether any stable variant can be established from the original culture. It is proposed that this *in vitro* cell line is suitable material for studying the biology of rare tumours such as rhabdomyosarcoma, as well as a source of cells which can legitimately be used as immunogen for raising and screening monoclonal antibodies to rhabdomyosarcoma.

This work was supported by the Imperial Cancer Research Fund. Julie Clayton was in receipt of a grant from the Child Health Research Appeal Trust at the Institute of Child Health. We wish to thank, J. Clarke, F. Gibson and L. Heath for their assistance in maintaining the cell lines and hybridomas used in this study. We also wish to thank Dr T. Sawada, (Kyoto, Japan), for supplying the rhabdomyosarcoma cell lines, and Mr G. Anderson for the EM studies. In addition we thank S. Watts for preparing and typing this manuscript.

### References

ABRAMSON, C.S., KERSEY, J.H. & LEBIEN, T.W. (1981). A monoclonal antibody (BA-1) reactive with cells of human B lymphocyte linneage. *J. Immunol.*, **126,** 83.

ALLAN, P.M., GARSON, J.A., HARPER, E.I. & 4 others (1983). Biological characterisation and clinical applications of a monoclonal antibody recognising an antigen restricted to neuroectodermal tissues. *Int. J. Cancer*, **31,** 591.

ALTMANSBERGER, M., WEBER, K., DROSTE, R. & OSBORN, M. (1985). Desmin specific marker for rhabdomyosarcomas of human and rat origin. *Am. J. Pathol.*, **118,** 85.

BEVERLEY, P.C.L. (1980). Production and use of monoclonal antibodies in transplant immunology. In *Proceedings of the 11th International Course on Transplant and Clinical Immunology*. p. 87. Exerpta Medica: Amsterdam.

BRODSKY, F.M., PARHAM, P., BARNSTABLE, C.J., CRUMPTON, M.J. & BODMER, W.F. (1979). Monoclonal antibodies for analysis of the HLA system. *Immunol. Rev.*, **47,** 3.

BRODSKY, F.M., PARHAM, P. & BODMER, W.F. (1980). Monoclonal antibodies to HLA monomorphic determinants. *Tissue Anal.*, **16,** 30.

DEBUS, E., WEBER, K. & OSBORN, M. (1983). Monoclonal antibodies to desmin, the muscle-specific intermediate filament protein. *EMBO J.*, **2,** 2305.

EISENBARTH, G.S., WALSH, F.S. & NIRENBERG, M. (1979). Monoclonal antibody to a plasma membrane antigen in neurons. *Proc. Natl Acad. Sci. USA*, **76,** 4913.

ENZINGER, F.M. & WEISS, S.W. (eds) (1983). *Soft Tissue Tumours*. p. 373. C.V. Mosby Co., St. Louis, Toronto, London.

GIARD, D.J., AARONSON, S.A., TODARO, G.J. & 4 others (1973). In vitro cultivation of human tumours: Establishment of cell lines derived from a series of solid tumours. *J. Natl Cancer Inst.*, **51,** 1417.

HURKO, O. & WALSH, F.S. (1983). Human foetal muscle specific antigen is restricted to regenerating myofibers in diseased adult muscle. *Neurology*, **33,** 734.

KEMSHEAD, J.T., FRITSCHY, J., ASSER, U., SUTHERLAND, R. & GREAVES, M.F. (1982). Monoclonal antibodies defining markers with apparent selectivity for particular haemopoietic cell types may also detect antigens on cells of neural crest origin. *Hybridoma*, **1,** 109.

KEMSHEAD, J.T., FRITSCHY, J., GOLDMAN, A., MALPAS, J.S. & PRITCHARD, J. (1983). Use of panels of monoclonal antibodies in the differential diagnosis of neuroblastoma and lymphoblastic disorders. *Lancet*, **i,** 12.

KEMSHEAD, J.T. & COAKHAM, H.B. (1983). The use of monoclonal antibodies for the diagnosis of intracellular malignancies and small round cell tumours of childhood. *J. Pathol.*, **4**, 249.

KENNETT, R.H. & GILBERT, F. (1979). Hybrid myelomas producing antibodies against a human neuroblastoma antigen present on fetal brain. *Science*, **203**, 1120.

KERSEY, J.H., LEBIEN, T.W., ABRAMSON, C.S. & 3 others (1981). P24: A human leukaemia associated and lympho hematopoietic progenitor cell surface structure identified with monoclonal antibody. *J. Exp. Med.*, **153**, 726.

LANE, E.B. (1982). Monoclonal antibodies provide specific intracellular markers for the study of epithelial tonofilament organisation. *J. Cell Biol.*, **92**, 665.

LEBIEN, T., KERSEY, J., NAKAZAWA, S., MINATO, K. & MINAWADA, J. (1982). Analysis of human leukaemia/lymphoma cell lines with monoclonal antibodies BA-1, BA-2, and BA-3. *Leukaemia Res.*, **6**, 299.

McALLISTER, R.M., MELNYK, J., FINKELSTEIN, J.Z., ADAMS, E.C. & GARDNER, M.B. (1969). Cultivation in vitro of cells derived from a human rhabdomyosarcoma. *Cancer*, **24**, 520.

McALLISTER, R.M., NICHOLSON, M., GARDNER, M.B. & 9 others. (1972). G type virus released from cultured human rhabdomyosarcoma cells. *Nature* (*New Biol*)., **235**, 3.

McALLISTER, R.M., NELSON-REES, W.A., PEER, M. & 5 others. (1975). Childhood sarcomas and lymphomas: Characterisation of new cell lines and search for Type-C virus. *Cancer*, **36**, 1804.

OSBORN, M. & WEBER, K. (1983). Biology of disease: Tumour diagnosis by intermediate filament typing: A novel tool for surgical pathology. *Lab Invest.*, **48**, 372.

OSBORN, (1984).

REYNOLDS, P.C., SMITH, G.R. & FRENKEL, E.P. (1981). The diagnostic dilemma of the small round cell neoplasm. *Cancer*, **48**, 2088.

SAVAGE, J.R.K. (1975). Classification and relationships of induced chromosomal structural changes. *J. Med. Genet.*, **12**, 103.

SUTOW, W.W., FERNBACH, D.J. & VIETTI, T.J. (1984). *Clinical Pediatric Oncology* (3rd Edition) C.V. Mosby, St. Louis, Toronto, 623.

TOKER, C. (1968). Embryonal rhabdomyosarcoma. An ultrastructural study. *Cancer*, **210**, 1164.

TRICHE, T.J. & ASKIN, F.B. (1983). Neuroblastoma and the differential diagnosis of small-, round-, blue-cell tumours. *Hum. Pathol.*, **14**, 569.

WOOD, J.N. & ANDERTON, B.H. (1981). Monoclonal antibodies to mammalian neurofilaments. *Biosci. Rep.*, **1**, 263.


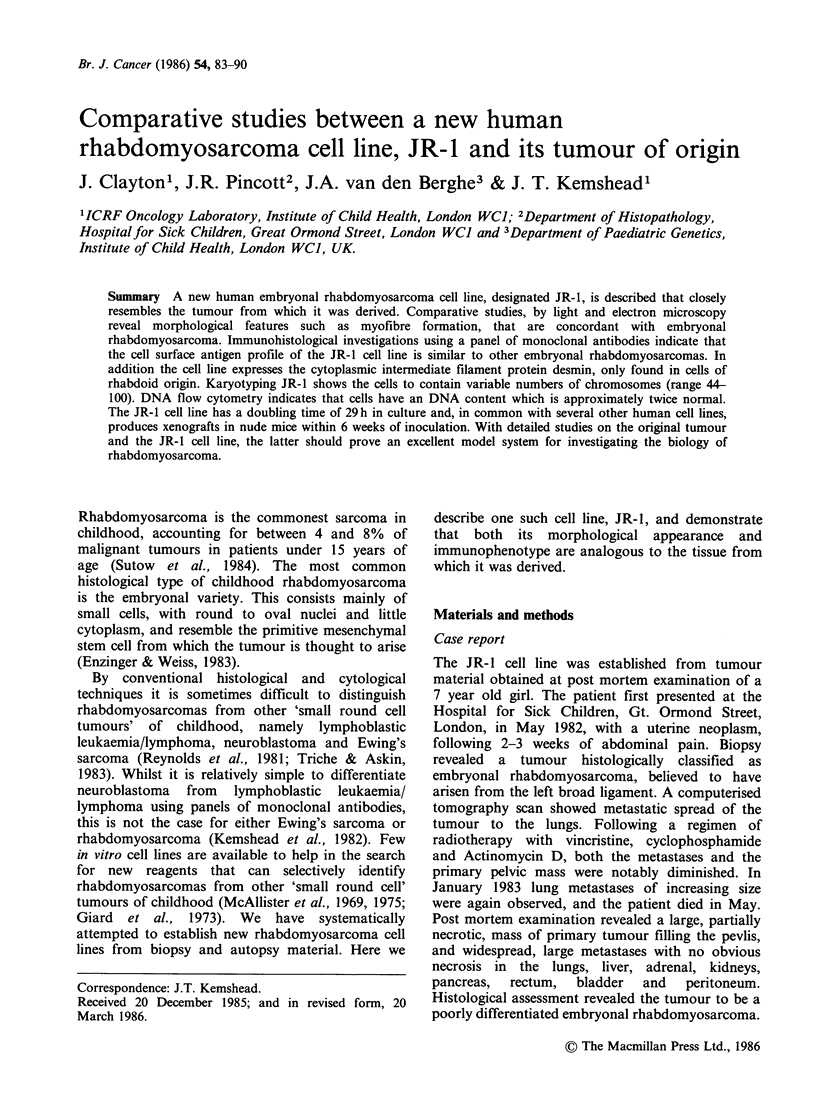

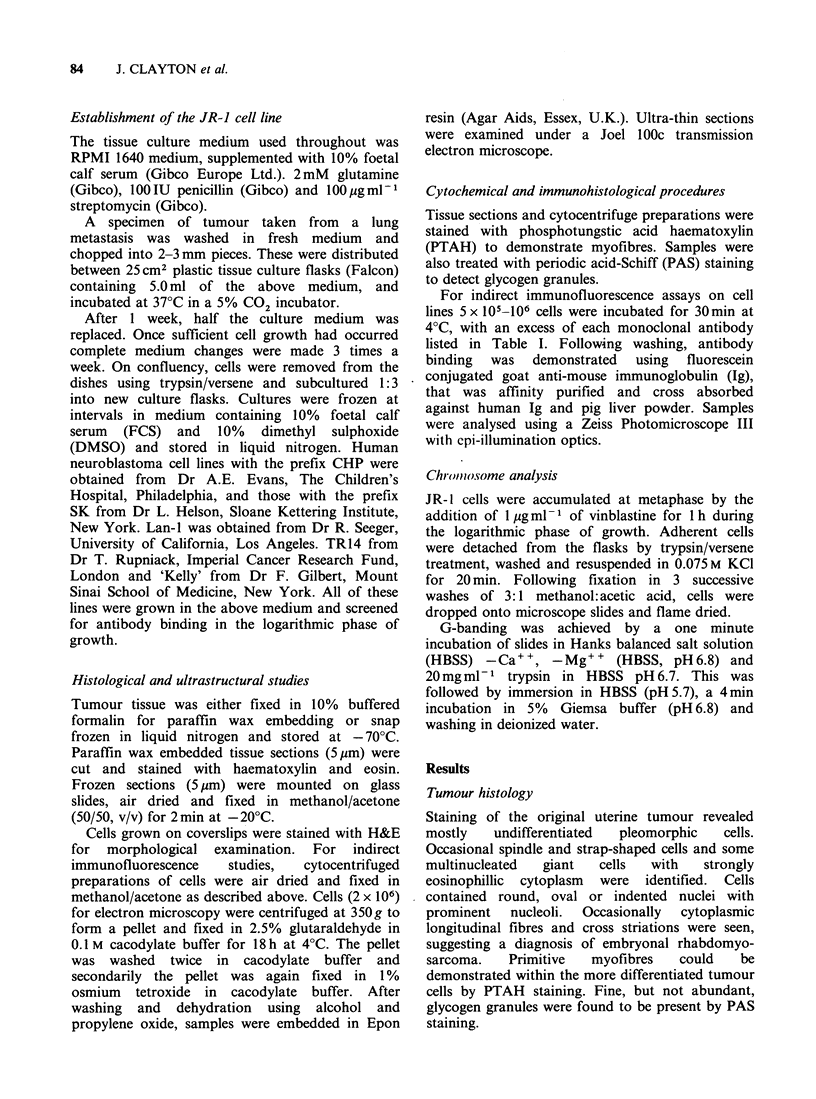

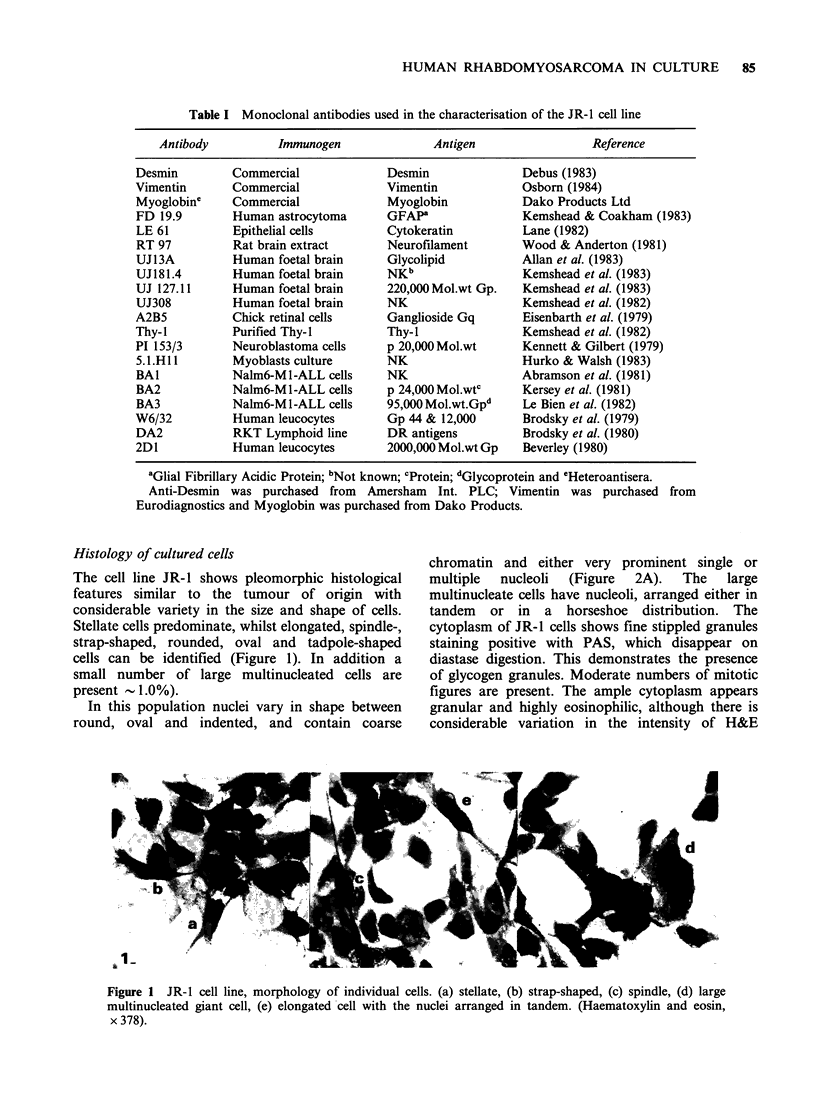

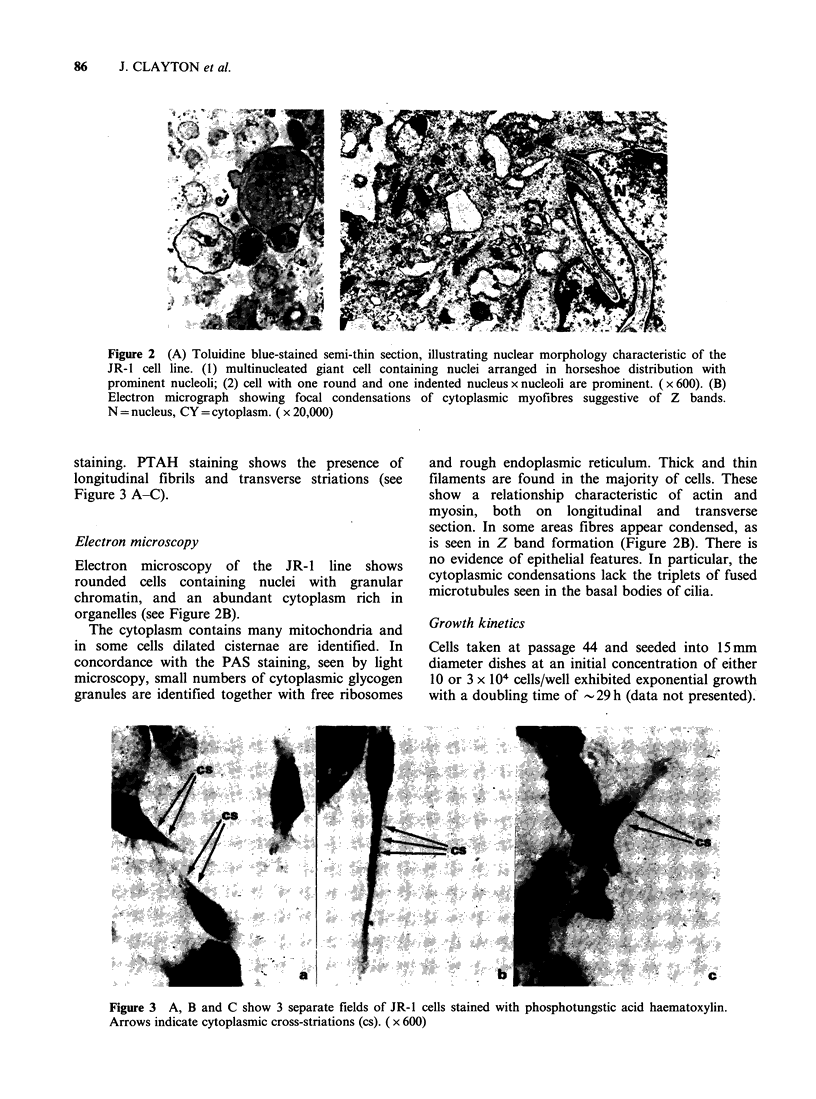

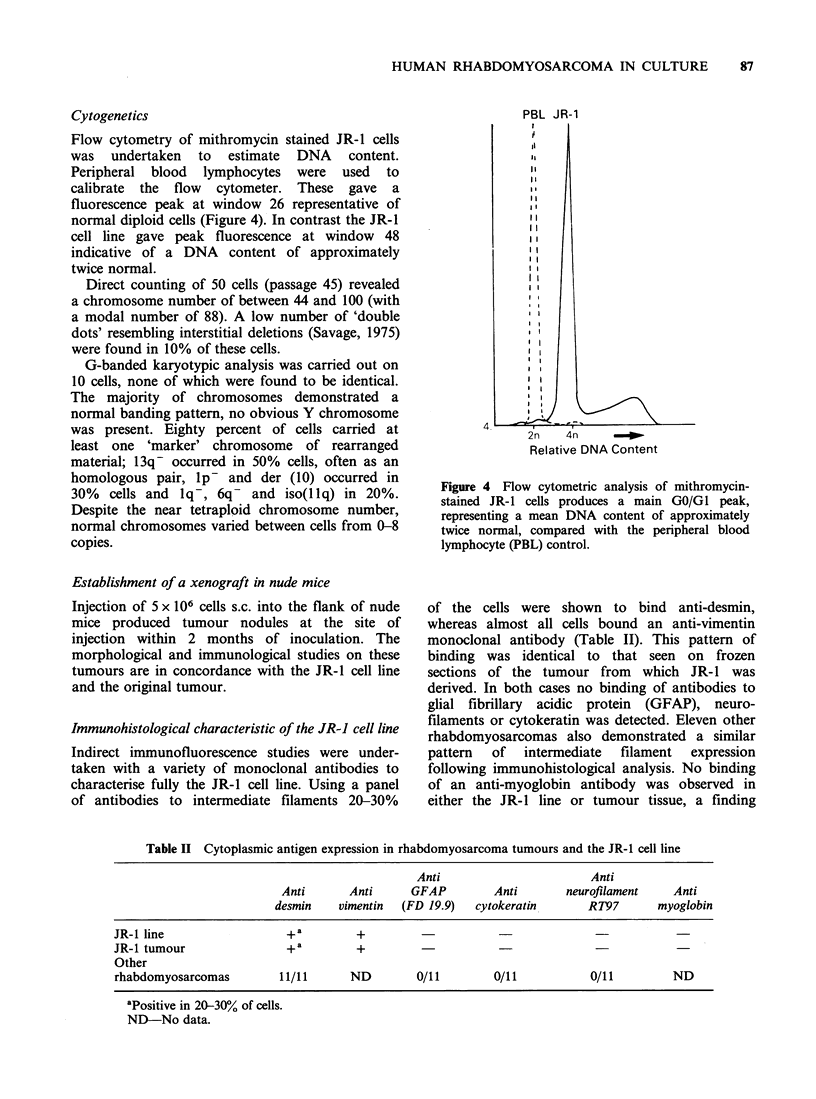

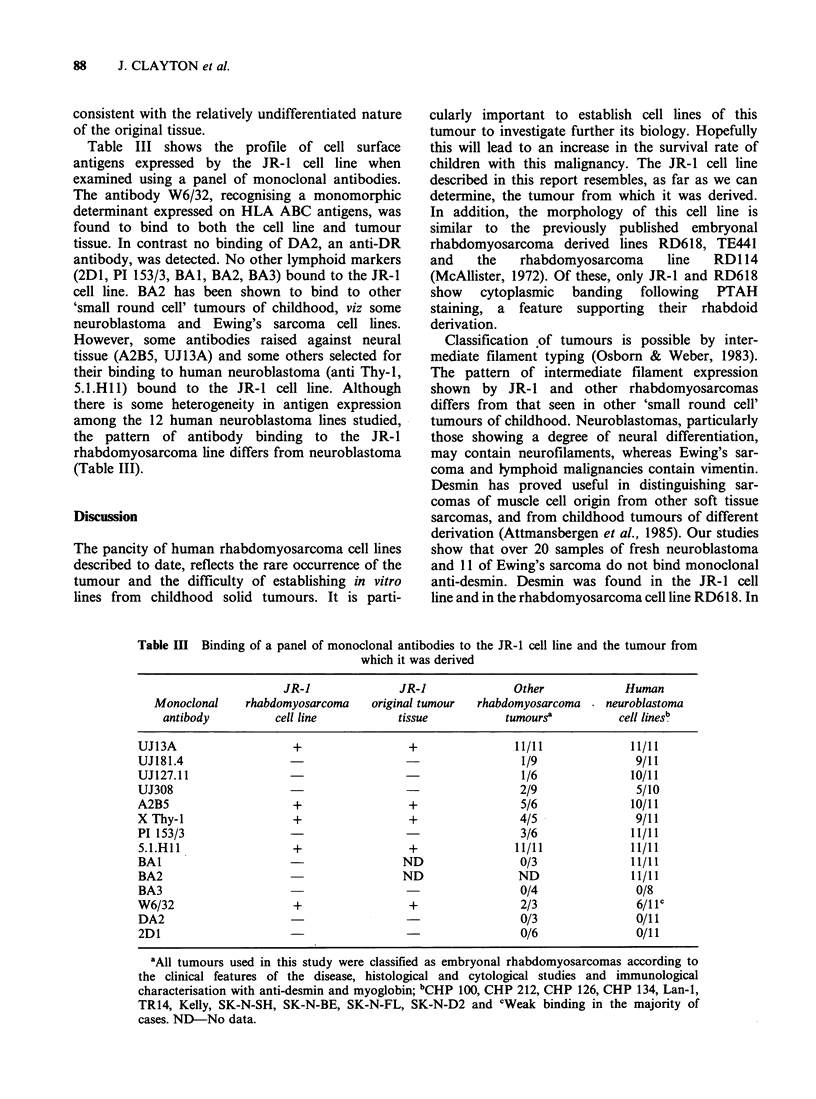

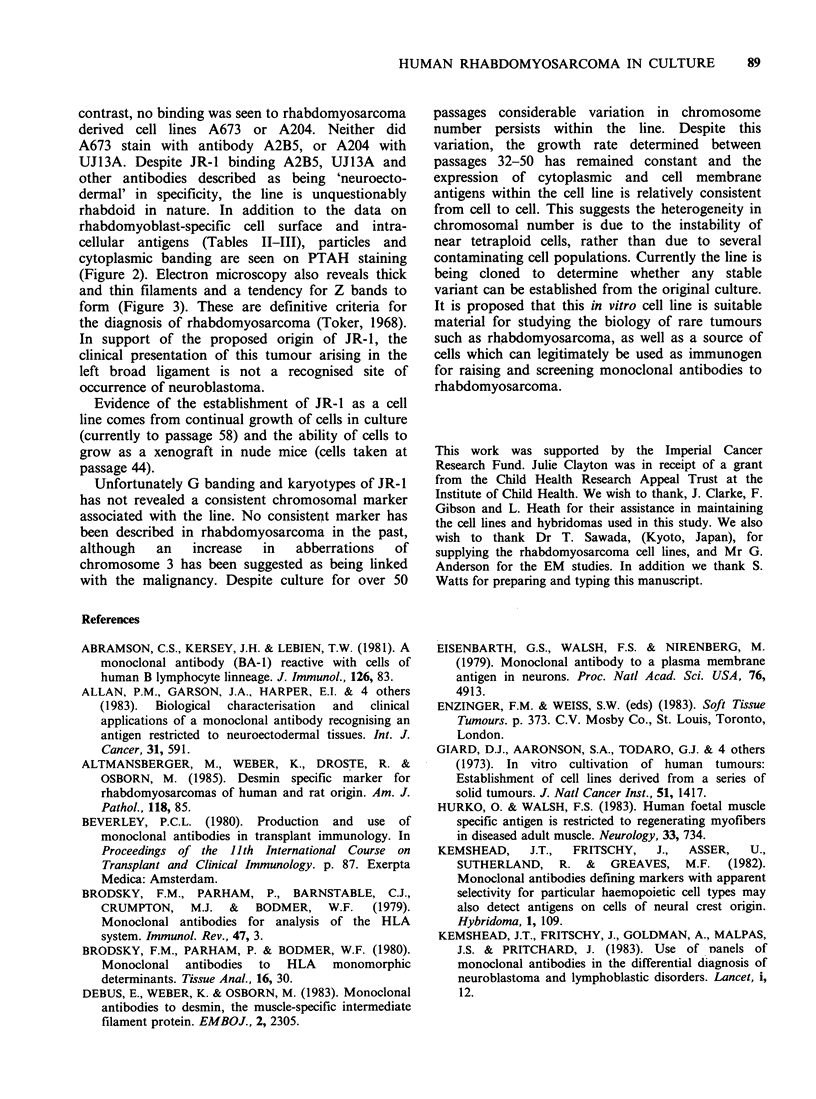

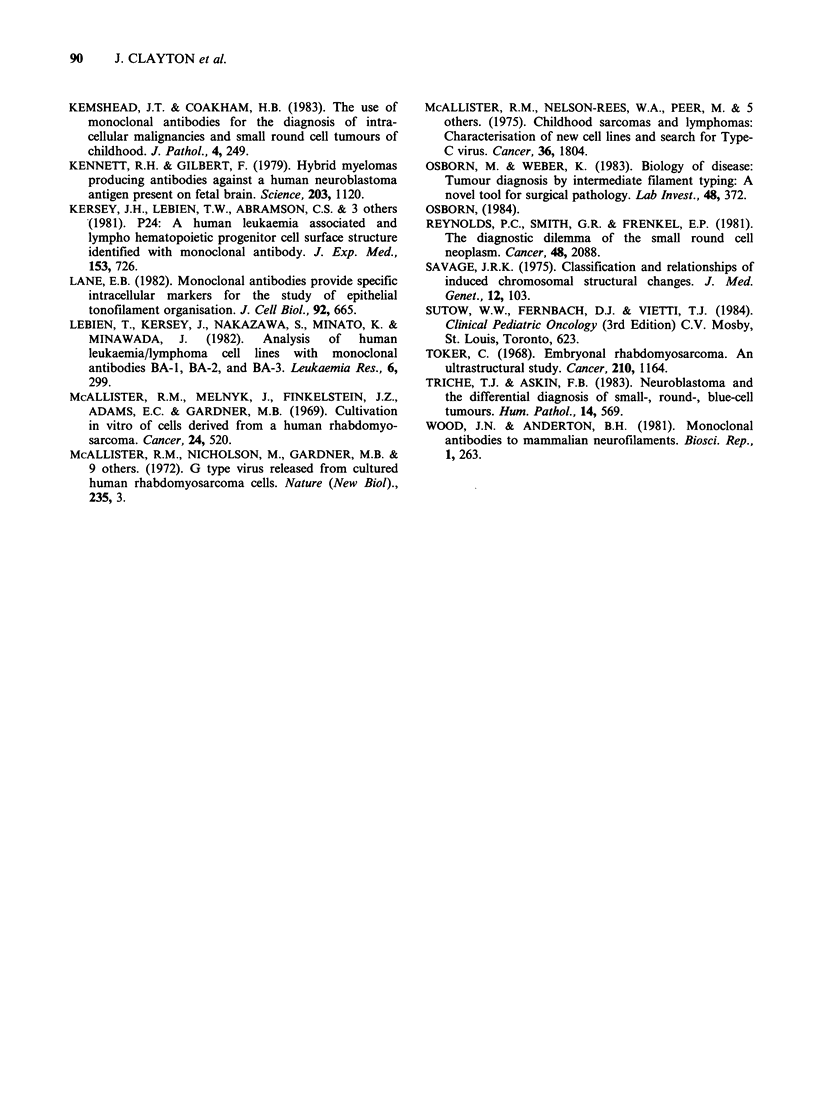


## References

[OCR_00595] Abramson C. S., Kersey J. H., LeBien T. W. (1981). A monoclonal antibody (BA-1) reactive with cells of human B lymphocyte lineage.. J Immunol.

[OCR_00600] Allan P. M., Garson J. A., Harper E. I., Asser U., Coakham H. B., Brownell B., Kemshead J. T. (1983). Biological characterization and clinical applications of a monoclonal antibody recognizing an antigen restricted to neuroectodermal tissues.. Int J Cancer.

[OCR_00607] Altmannsberger M., Weber K., Droste R., Osborn M. (1985). Desmin is a specific marker for rhabdomyosarcomas of human and rat origin.. Am J Pathol.

[OCR_00620] Brodsky F. M., Parham P., Barnstable C. J., Crumpton M. J., Bodmer W. F. (1979). Monoclonal antibodies for analysis of the HLA system.. Immunol Rev.

[OCR_00626] Brodsky F. M., Parham P., Bodmer W. F. (1980). Monoclonal antibodies to HLA--DRw determinants.. Tissue Antigens.

[OCR_00631] Debus E., Weber K., Osborn M. (1983). Monoclonal antibodies to desmin, the muscle-specific intermediate filament protein.. EMBO J.

[OCR_00636] Eisenbarth G. S., Walsh F. S., Nirenberg M. (1979). Monoclonal antibody to a plasma membrane antigen of neurons.. Proc Natl Acad Sci U S A.

[OCR_00647] Giard D. J., Aaronson S. A., Todaro G. J., Arnstein P., Kersey J. H., Dosik H., Parks W. P. (1973). In vitro cultivation of human tumors: establishment of cell lines derived from a series of solid tumors.. J Natl Cancer Inst.

[OCR_00675] Kemshead J. T., Coakham H. B. (1983). The use of monoclonal antibodies for the diagnosis of intracranial malignancies and the small round cell tumours of childhood.. J Pathol.

[OCR_00658] Kemshead J. T., Fritschy J., Asser U., Sutherland R., Greaves M. F. (1982). Monoclonal antibodies defining markers with apparent selectivity for particular haemopoietic cell types may also detect antigens on cells of neural crest origin.. Hybridoma.

[OCR_00666] Kemshead J. T., Goldman A., Fritschy J., Malpas J. S., Pritchard J. (1983). Use of panels of monoclonal antibodies in the differential diagnosis of neuroblastoma and lymphoblastic disorders.. Lancet.

[OCR_00681] Kennett R. H., Gilbert F. (1979). Hybrid myelomas producing antibodies against a human neuroblastoma antigen present on fetal brain.. Science.

[OCR_00686] Kersey J. H., LeBien T. W., Abramson C. S., Newman R., Sutherland R., Greaves M. (1981). P-24: a human leukemia-associated and lymphohemopoietic progenitor cell surface structure identified with monoclonal antibody.. J Exp Med.

[OCR_00693] Lane E. B. (1982). Monoclonal antibodies provide specific intramolecular markers for the study of epithelial tonofilament organization.. J Cell Biol.

[OCR_00698] LeBien T., Kersey J., Nakazawa S., Minato K., Minowada J. (1982). Analysis of human leukemia/lymphoma cell lines with monoclonal antibodies BA-1, BA-2 and BA-3.. Leuk Res.

[OCR_00705] McAllister R. M., Melnyk J., Finkelstein J. Z., Adams E. C., Gardner M. B. (1969). Cultivation in vitro of cells derived from a human rhabdomyosarcoma.. Cancer.

[OCR_00717] McAllister R. M., Nelson-Rees W. A., Peer M., Laug W. E., Isaacs H., Gilden R. V., Rongey R. W., Gardner M. B. (1975). Childhood sarcomas and lymphomas. Characterization of new cell lines and search for type-C virus.. Cancer.

[OCR_00723] Osborn M., Weber K. (1983). Tumor diagnosis by intermediate filament typing: a novel tool for surgical pathology.. Lab Invest.

[OCR_00729] Reynolds C. P., Smith R. G., Frenkel E. P. (1981). The diagnostic dilemma of the "small round cell neoplasm": catecholamine fluorescence and tissue culture morphology as markers for neuroblastoma.. Cancer.

[OCR_00744] Toker C. (1968). Embryonal rhabdomyosarcoma. An ultrastructural study.. Cancer.

[OCR_00748] Triche T. J., Askin F. B. (1983). Neuroblastoma and the differential diagnosis of small-, round-, blue-cell tumors.. Hum Pathol.

[OCR_00753] Wood J. N., Anderton B. H. (1981). Monoclonal antibodies to mammalian neurofilaments.. Biosci Rep.

